# Cochlear Implantation in a Case of Relapsing Polychondritis With Profound Hearing Loss and Labyrinthine Ossification

**DOI:** 10.7759/cureus.55031

**Published:** 2024-02-27

**Authors:** Abhijeet Mishra, Preetam Chappity, Sanjay K Behera, Mohnish Grover, Gaurav Gupta

**Affiliations:** 1 Otolaryngology, All India Institute of Medical Sciences, Bhubaneswar, Bhubaneswar, IND; 2 Otolaryngology, Sawai Man Singh (SMS) Medical College, Jaipur, IND; 3 Otolaryngology, Sarder Patel Medical College, Bikaner, IND

**Keywords:** scala vestibuli insertion, labyrinthitis ossificans, hearing loss, cochleostomy, labyrinthine ossification, relapsing polychondritis, cochlear implant

## Abstract

Relapsing polychondritis is an autoimmune disorder causing inflammation of cartilaginous structures, sensory epithelium, and cardiovascular system. Hearing loss is a rare and dreadful complication of this pathology. We report a case of relapsing polychondritis in a 38-year-old female who developed gradually progressive bilateral profound hearing loss. She did not have any improvement with medical management. Cochlear implantation was performed to rehabilitate her hearing. As the scala tympani was obliterated, a scala vestibuli insertion was performed. A complete insertion was possible with a compressed electrode, and she had good evoked compound action potential scores. Her categories of auditory performance scores were 6 at the end of one year. Patients with relapsing polychondritis can progress to profound hearing loss in rare cases and should be carefully followed up to identify early labyrinthine ossification. A scala vestibuli insertion can be performed with good outcomes in cases with ossification involving scala tympani. The surgeon should be ready for a middle-turn cochleostomy or a drill-out procedure in patients with advanced ossification.

## Introduction

Relapsing polychondritis (RP), though rare, can sometimes lead to profound sensorineural hearing loss (SNHL) requiring cochlear implant surgery. It is an autoimmune condition characterized by inflammation of the cartilaginous structures, sensory organs, and the cardiovascular system. Jaksch-Wartenhorst first described it in 1923. It is diagnosed primarily by clinical evaluation as specific biological markers are not available. McAdam criteria are widely used for diagnosing RP, which requires three or more of the following clinical signs: recurrent chondritis of the pinna, nasal chondritis, non-erosive arthritis, scleritis/uveitis, chondritis of the respiratory tract, and cochlear and/or vestibular dysfunction, along with histological confirmation [1]. Damiani and Levine have further revised this criterion [2]. 

Approximately 40-50% of patients have inner ear disorders; however, progression to profound hearing loss is rare [3]. Cochlear implantation might be the only option for hearing rehabilitation in such cases, but the associated labyrinthine ossification creates a hurdle for successful implantation [4,5].

In this report, we will discuss our experience in managing a patient of RP with rapidly progressing bilateral SNHL and bilateral cochlear ossification. Our patient had a progressive hearing loss in both ears over a period of two years. Besides hearing loss, other symptoms of the disease spectrum, like scleritis and polyarthritis, impaired her quality of life significantly.

## Case presentation

A female patient in her late 30s was referred to our center with complaints of progressive bilateral hearing loss. She was diagnosed with RP based on the presence of recurrent scleritis, polyarthritis, and progressive hearing loss in both ears, fulfilling the Damiani and Levine criteria [2]. The symptoms had started four years back, with bilateral scleritis being the earliest symptom, followed by polyarthritis involving the elbows, wrists, fingers, and knee joints of both sides. Hearing loss started two years later in the left ear first, followed by the right ear. The hearing loss gradually progressed over two years. She didn’t complain of tinnitus or vestibular symptoms. Based on her symptomatology, she was started on systemic steroids and immunomodulators (cyclophosphamide and azathioprine), which helped relieve the scleritis and arthritis. There was no improvement in the hearing loss. Her general condition improved after the treatment, but the hearing loss progressed until she had complete deafness in both ears. 

**Figure 1:**
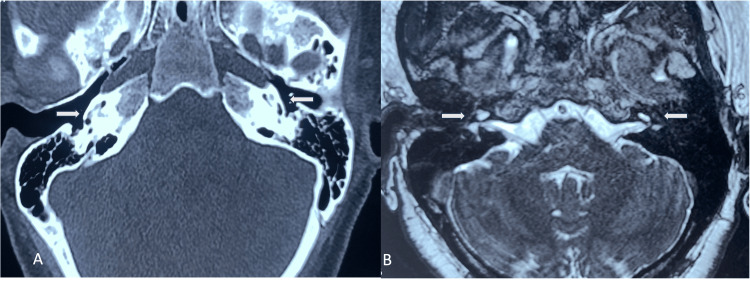
Radiology of the temporal bone showing ossification of the cochlea A: High-resolution CT of temporal bone showing the presence of partial ossification (white arrow) involving the basal turn of cochlea on the right side and both basal and middle turn on the left side. B: T2 weighted image of MRI scan showing faint fluid signal (white arrow) in the basal turn of left cochlea and middle and apical turns on the right side

Pure tone audiometry showed profound hearing loss in both ears. Brainstem-evoked response audiometry (BERA) showed a lack of waves at 90db, and the otoacoustic emission (OAE) showed bilateral “REFER”. Vestibular function tests showed no abnormality. Hearing aids were tried initially with no benefit. Radiological investigations were done in the form of a high-resolution computed tomography (HRCT) of the temporal bone and magnetic resonance imaging (MRI) of the inner ear and brain (Figure 1).

The basal, apical, and middle turns of the cochlea were partially ossified on both sides, as were the superior, posterior, and lateral semicircular canals, with evidence of more ossification on the left side compared to the right. Similar findings were noted on MRI with faint hyperintense signals in both cochlea's apical and middle turns, right more than the left. The vestibulocochlear nerve was normal on both sides. 

**Figure 2:**
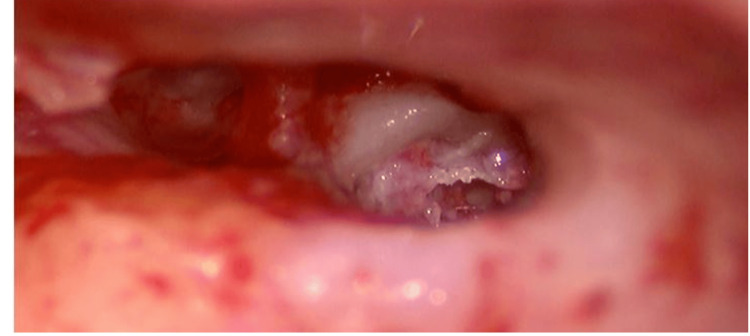
Fibrous tissue seen through posterior tympanotomy obliterating the round window and extending into the basal turn of cochlea

As the patient could only afford single-sided implantation, we selected the right ear to gain maximum benefit from the implantation. A FORM 19 electrode (MED-EL, Innsbruck, Austria) with 24 electrodes/ 12 channels was used for implantation. We approached the round window via the facial recess approach. The round window was obliterated, and whitish fibrous tissue was seen obliterating it (Figure 2).

**Figure 3:**
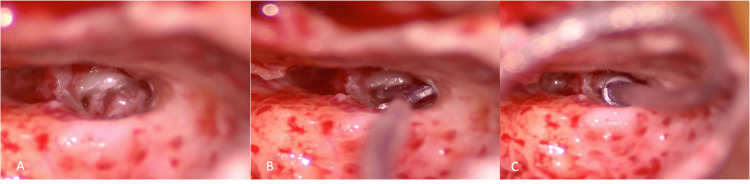
Intraoperative images of scala vestibuli insertion of the implant A: Cochlesotomy was done anterosuperior to round window to identify the lumen of scala vestubli B: A dummy electrode/depth gauge was inserted to confirm the patency of the scala vestibuli C: Complete insertion of the electrode was performed

Drilling was done, and fibrous tissue was removed to identify the obliterated round window. It was further drilled antero-inferiorly to locate any lumen in the scala tympani. The scala tympani was found to be wholly obliterated with fibrosis. The superior strut of bone was drilled, and the oval window was exposed to prepare for either a scala vestibuli insertion or a middle-turn cochleostomy. A cochleostomy was performed anterosuperior to the round window and anteroinferior to the oval window. The lumen of scala vestibuli was identified, and the patency was confirmed using a depth gauge (Figure 3).

**Figure 4:**
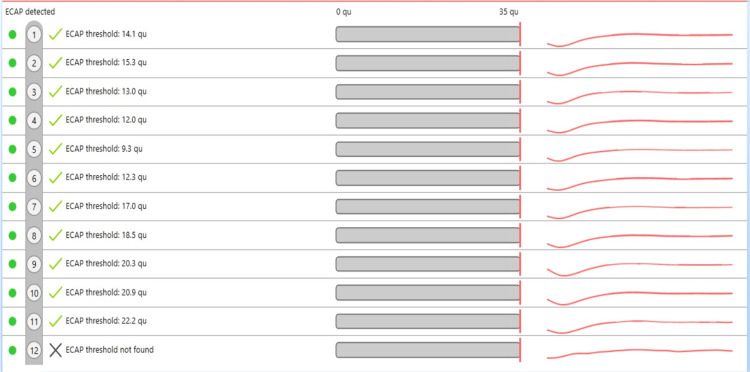
The intraoperative auto neural response imaging was obtained for 11 channels and the 12th channel response was noted on manual testing

The implant was then inserted completely. The response was confirmed by neural response imaging (NRI) (Figure 4).

**Figure 5:**
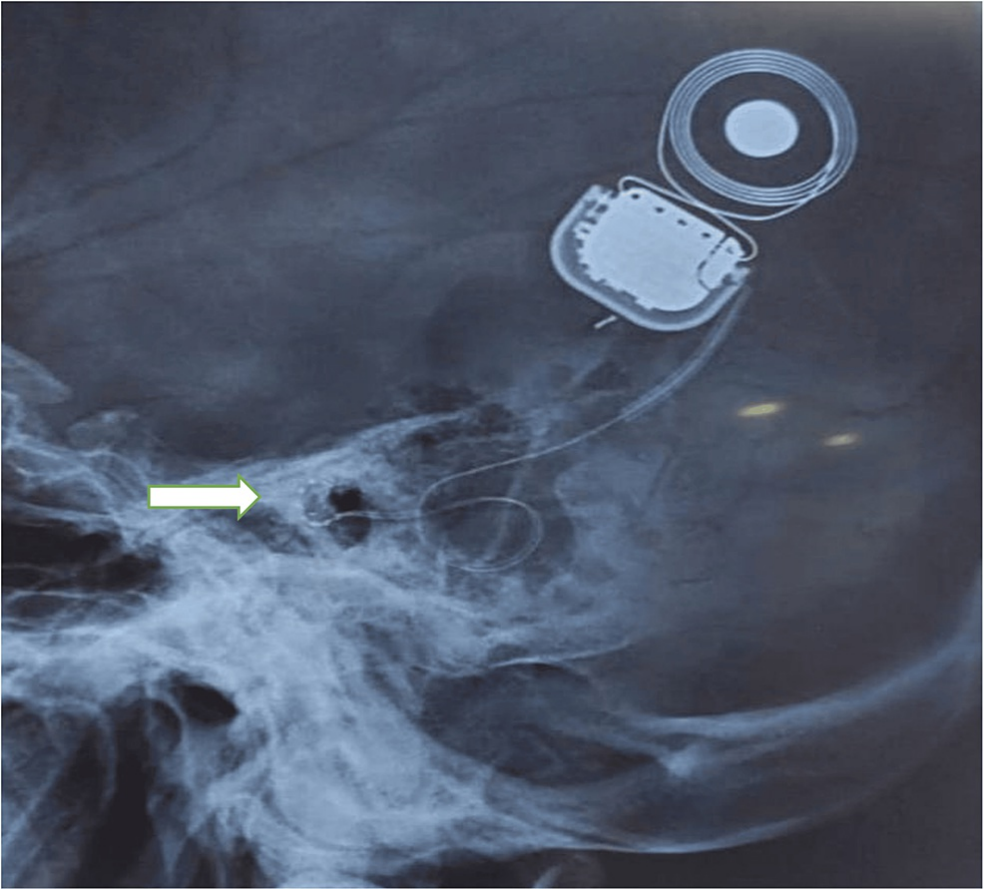
Postoperative X-ray image confirming the presence of electrode in the cochlea (white arrow)

The position of the electrodes was also confirmed using an X-ray (Figure 5).

The post-operative period was uneventful. The patient responded well at the switch-on, being aware of environmental sounds. Speech evaluation done one year after surgery showed a CAP (categories of auditory performance) score of 6, implying an understanding of speech without lip reading. 

## Discussion

RP is a multisystem disorder, and clinical features include nasal chondritis, recurrent auricular chondritis, non-erosive polyarthritis, laryngotracheal symptoms, ocular inflammation, audiovestibular symptoms, and cardiovascular disease symptoms [6]. Hearing loss in RP can be conductive or sensory neural type. Conductive deafness may be due to external auditory canal/pinna edema and collapse [7,8]. The pathophysiology of SNHL in RP is yet to be fully understood [8]. Miyazawa et al. found that hearing loss was associated with the specific degeneration of type II collagen-enriched tectorial membrane by immunological staining of type II collagen in pathological temporal bone preparations in patients with RP [9]. Serum anti-labyrinthine antibodies were detected in an RP patient with audiovestibular dysfunction by Issing et al. [10]. Schuknecht, in his study, reported that the cartilage in the inner ear was absent, implying obliterative vasculitis of the labyrinthine artery or its branches [11].

Clinical criteria for diagnosis were first described by McAdam, which was later revised by Damiani and Levine and necessitate one of the following for the diagnosis: “(i) at least three or more of McAdam’s criteria without the need for specific histologic findings, (ii) one or more of McAdam’s signs with positive histologic confirmation, and (iii) chondritis of two or more separate anatomic sites with response to steroids and/or dapsone” [2]. Our patient met the first criteria given by Damiani and Levine. SNHL in patients with RP usually does not progress to profound hearing loss and immunosuppressive drugs can halt the progression of hearing loss in most patients [12]. However, there was a rapid progression of hearing loss despite early diagnosis and treatment with immunosuppressive drugs (systemic steroids, cyclophosphamide, and azathioprine) in our patient.

Cochlear implantation has shown promising results in immune-mediated inner ear diseases (IMIED) like systemic lupus erythematosus, Cogan's syndrome, and Buerger’s disease [13]. Pathogenesis of RP is very similar to IMIED; thus, cochlear implants can be effective for cochlear dysfunction in patients with RP. Seo et al. first reported cochlear implantation in RP with good hearing results on follow-up [14]. 

However, cochlear implant surgery can prove to be challenging in patients with RP due to their common association with labyrinthitis ossificans [4,5]. Cochlear ossification was previously considered a relative contraindication for cochlear implant surgery. With novel surgical techniques and the ever-changing technology of the electrode arrays, it is now possible to do implantation in such cases [15]. A scala vestibuli approach is a valuable option for obtaining electrode insertion. It was described by Steenerson et al. in a partially ossified cochlea [16]. In cases of cochlear ossification, scala vestibuli is often patent, making it an excellent alternative to the conventional scala tympani approach [17]. However, this approach has shortcomings, like the need for higher current levels, increased vestibular stimulation, and more trauma during insertion [18]. In their retrospective study, Trudel et al. concluded that this approach provides comparable auditory performance to the scala tympani approach. Impedance values were higher in the scala vestibuli group, but no deleterious effects on programming parameters were noted. Also, being technically easier, it can be used as the primary surgical approach in ossified scala tympani [19]. In the case of obliteration of scala vestibuli, a middle-turn cochleostomy can be performed with an anterograde or a retrograde insertion, or a drill-out procedure can be tried as a last resort.

## Conclusions

Patients with RP can, in rare cases, progress to profound hearing loss and should be carefully followed up to identify early labyrinthine ossification. A scala vestibuli insertion with a compressed array can be performed with good outcomes in cases with ossification involving scala tympani. The surgeon should be ready for a middle-turn cochleostomy or a drill-out procedure in patients with advanced ossification.
